# Towards a holistic epidemiology of *Streptococcus agalactiae* using the BakRep repository

**DOI:** 10.3389/fbinf.2026.1924154

**Published:** 2026-09-30

**Authors:** Linda Fenske, Oliver Schwengers, Alexander Goesmann

**Affiliations:** Bioinformatics and Systems Biology, Justus Liebig University Giessen, Giessen, Germany

**Keywords:** antimicrobial resistance, capsular serotypes, clonal complexes, GWAS, metadata, multilocus sequence typing, population structure, *Streptococcus agalactiae*

## Abstract

**Background:**

*Streptococcus agalactiae* is a versatile multi-host pathogen that can cause severe neonatal disease in humans and mastitis in dairy animals. Its ability to infect a wide range of hosts is largely driven by its high genomic plasticity and the acquisition of distinct accessory genes. The global population of *S. agalactiae* is characterized by multiple capsular serotypes and clonal complexes that differ in their propensity to cause invasive disease, including hypervirulent CC17 (often serotype III) associated with neonatal meningitis, whereas CC1/CC19/CC23 are more often colonizing lineages. Although widely studied, most research is limited to particular regions or single outbreak events, offering only fragmented snapshots rather than a comprehensive global picture.

**Methods:**

To move beyond region- or outbreak-limited studies, this work has analyzed 37970 *S. agalactiae* genomes from BakRep, integrating serotypes, MLST, AMR genes, lineage-specific genes, and descriptive metadata to map current trends and identify potential gaps in public data.

**Results:**

The dataset largely matched the known population structure with serotype III, Ia, and V most common and stable serotype/clonal complex lineages (e.g., III-2/CC17, Ia/CC23, CC1/V), while also increasing serotype diversity. Lineages differed in their accessory-gene profiles, with III-2/CC17 being enriched for virulence and adhesion genes, while other groups showed either greater genomic plasticity (mobile/phage genes) or niche specialization. AMR was widespread with very high tetracycline resistance (>80%), frequent MLSB resistance determinants, and emerging aminoglycoside resistance in some genomes.

**Conclusions:**

Overall, it became evident that the associated metadata contained substantial gaps. Missing or incomplete information limits biological interpretation, pointing out the importance of rigorously curated, structured metadata for maximizing the value of ongoing sequencing efforts.

## Introduction

*Streptococcus agalactiae*, also known as Group B *Streptococcus* (GBS), is a Gram-positive bacterium capable of causing infections in various host species. In human medicine, GBS is a common cause of neonatal infections, primarily transmitted from mother to newborn during childbirth (Le Doare and Heath, 2013). An estimated 20%–30% of pregnant women are colonized with GBS, with around 50% of their newborns acquiring colonization and approximately 1% developing invasive disease (Jones et al., 2006; Bergeron et al., 2000). Asymptomatic GBS colonization is also commonly observed in both adults (Manning et al., 2004) and elderly individuals (Sendi et al., 2008). In the veterinary context, GBS is primarily known as a contagious udder pathogen linked to mastitis. While it is most commonly associated with mastitis in cattle, cases have also been reported in other dairy species, including goats (Danmallam and Pimenov, 2019; Shi et al., 2022), sheep (Ozavci et al., 2023; ΖΔΡΑΓΚΑΣ et al., 2017), and camels (Crestani et al., 2022). Although most published GBS research centers on human and bovine cases, GBS has also been detected in several other terrestrial (Fenske et al., 2025; Shuster et al., 2013) and aquatic species (Delannoy et al., 2013; Numberger et al., 2021; Abdulrahim et al., 2026). The broad host range of GBS has been linked to its high genomic plasticity, which enables integration of accessory genes into its chromosome, allowing strains to colonize diverse hosts and ecological niches, providing an evolutionary advantage (Richards et al., 2019).

The outer layer of GBS consists of a polysaccharide capsule, whose antigenic properties serve as the basis for further subclassification into currently ten major serotypes (Ia, Ib, II-IX) (Wilkinson and Moody, 1969) and four subtypes of serotype III (III-1, III-2, III-3, III-4) (Kong et al., 2002). The prevalence and distribution of serotypes are known to vary depending on host species, virulence levels, or geographic region (Bianchi-Jassir et al., 2020). Additionally, multilocus sequence typing (MLST) classifies GBS isolates into distinct sequence types (STs), which can be further grouped into clonal complexes (CCs). This can offer insights into the pathogenicity of isolates. For example, CC17 is known as a hyper-virulent cluster, increasingly associated with neonatal infections (Teatero et al., 2016; Jamrozy et al., 2020). Specifically, the combination of serotype III and CC17 is considered responsible for the majority of neonatal meningitis cases (Hsu et al., 2021), likely due to specific genes encoding secreted and surface proteins that facilitate interactions between the bacterium and host cells (Teatero et al., 2016). In contrast, CC1, CC19, and CC23 are common colonizers of pregnant women and appear well adapted to the vaginal mucosa, with relatively limited invasive potential in neonates (Manning et al., 2008; Teatero et al., 2017). In general, antibiotic resistance (AMR) is also a growing concern in GBS strains. Intrapartum antibiotic prophylaxis (IAP) is administered to pregnant women based on risk factors or screening guidelines, making continuous monitoring of resistance rates essential (Hayes et al., 2020). In mastitis cases, particularly in middle-income countries with higher GBS prevalence and widespread antimicrobial use, including broad-spectrum and critically important antibiotics, AMR in multi-host pathogens reduces treatment options for both animals and humans (Oliveira et al., 2022).

Numerous studies have analyzed GBS, though they are often restricted to specific geographic regions or isolated outbreak events. As a result, only limited subsets are analyzed rather than a more holistic overview of the entire population. In 2024, BakRep was released (Fenske et al., 2024), featuring extensive genome characterizations based on 661,405 uniformly processed assemblies of all short-read sequence data from the European Nucleotide Archive (ENA) up to November 2018 (Blackwell et al., 2021). To date, the collection of uniformly assembled data has been expanded to 2440377 assemblies (Hunt et al., 2024), which have since been characterized and made accessible through the BakRep web repository. With its vast collection of genomic information and associated metadata, this repository provides an ideal resource for obtaining a comprehensive overview of publicly available sequence data, enabling insights beyond individual studies.

In the present study, we conducted a comparative analysis of 37,970 GBS genomes contained in BakRep v2. We characterized all isolates by MLST, capsular serotypes, AMR genes, and identified putative lineage-specific and accessory genes (including mobile genetic elements, heavy-metal resistance genes or virulence genes). This was complemented with metadata on geographic origin, host species, and disease patterns. This integrated analysis helps evaluate current trends in the GBS study landscape, highlight well-studied areas, and identify understudied gaps with only scarce public data.

## Methods

### Export data from the BakRep repository

All genome data and associated metadata were retrieved from BakRep (https://bakrep.computational.bio/; retrieved: 17.06.2025) (Fenske et al., 2024). For this, the repository was searched for entries where the species exactly matches “*Streptococcus* agalactiae” (operator “eq”) and the summary of the results were exported into a TSV file. Additional files provided by BakRep (mlst profiles, annotations, FASTA files) were downloaded via the BakRep CLI tool (https://github.com/ag-computational-bio/bakrep-cli) by providing the dataset IDs of the identified GBS genomes. The sole inclusion criterion was an exact species match to *S. agalactiae* in BakRep. No exclusion criteria based on assembly quality or metadata completeness were applied. This was a deliberate decision, as the aim of the study was to evaluate the public data resource in its current state rather than to generate a refined dataset for downstream population-genomic inference. Accordingly, all 37,970 genomes classified as *S. agalactiae* were included in the overall dataset.

### Data analysis

Capsular serotypes were determined using KMA (v1.4.12) using the FASTA files as input (Clausen et al., 2018). AMR genes were identified using AMRFinderPlus (v4.0.23) with database version 2024-12–18.1. (Feldgarden et al., 2019). CCs were assigned according to the PubMLST database (https://pubmlst.org/bigsdb?db=pubmlst_sagalactiae_seqdef&page=downloadProfiles&scheme_id=1; retrieved: 02.11.2025). Bakta-generated GFF3 annotation files (Schwengers et al., 2021) were used as input for Panaroo (v1.5.0) (Tonkin-Hill et al., 2020). The resulting gene presence/absence matrix, combined with a binary trait file of selected phenotypes, was then used to run a pan-GWAS in scoary2 (v0.0.15) using default settings (Roder et al., 2024). Scoary2 analyses were performed on the five most common serotype/clonal-complex combinations (Ia/23, II/22, III-2/17, IV/459, V/1) to identify genes predominantly associated with each combination. Genes identified as significantly associated with a given serotype/clonal-complex combination were subsequently characterized based on their functional annotation as provided by Bakta. For the creation of the phylogenetic tree, mashtree (v1.4.6) was used (Katz et al., 2019). For visualization of the phylogeny, iTol (v7) was used (Letunic and Bork, 2021). The metadata retrieved from BakRep and all additional results have been analyzed using R (v4.4.2).

## Results

### Sequence statistics of GBS genomes

BakRep v2 contained 37,970 genomes classified as *S. agalactiae*. The average estimated completeness genomes was 99.94%, with only 98 (0.26%) genomes below 99%. The average estimated contamination was 0.49%. 139 (0.37%) genomes showed contamination below 50% and 529 (1.39%) below 1% (Supplementary Figure S1). Assembled contigs ranged in length from 105,259 to 12119634 base pairs (bp), with a mean genome size of 2095432 bp. GBS genomes exceeding 2.6 Mbp are flagged as unusually large in the NCBI database. 206 genomes exceeding this threshold and also showed markedly higher estimated contamination (data not shown). The GC content ranged from 33.25% to 58.16% (mean 35.33%) and the number of contigs per assembly ranged from 10 to 6,016 (mean 51.59) (Figure 1).

**FIGURE 1:**
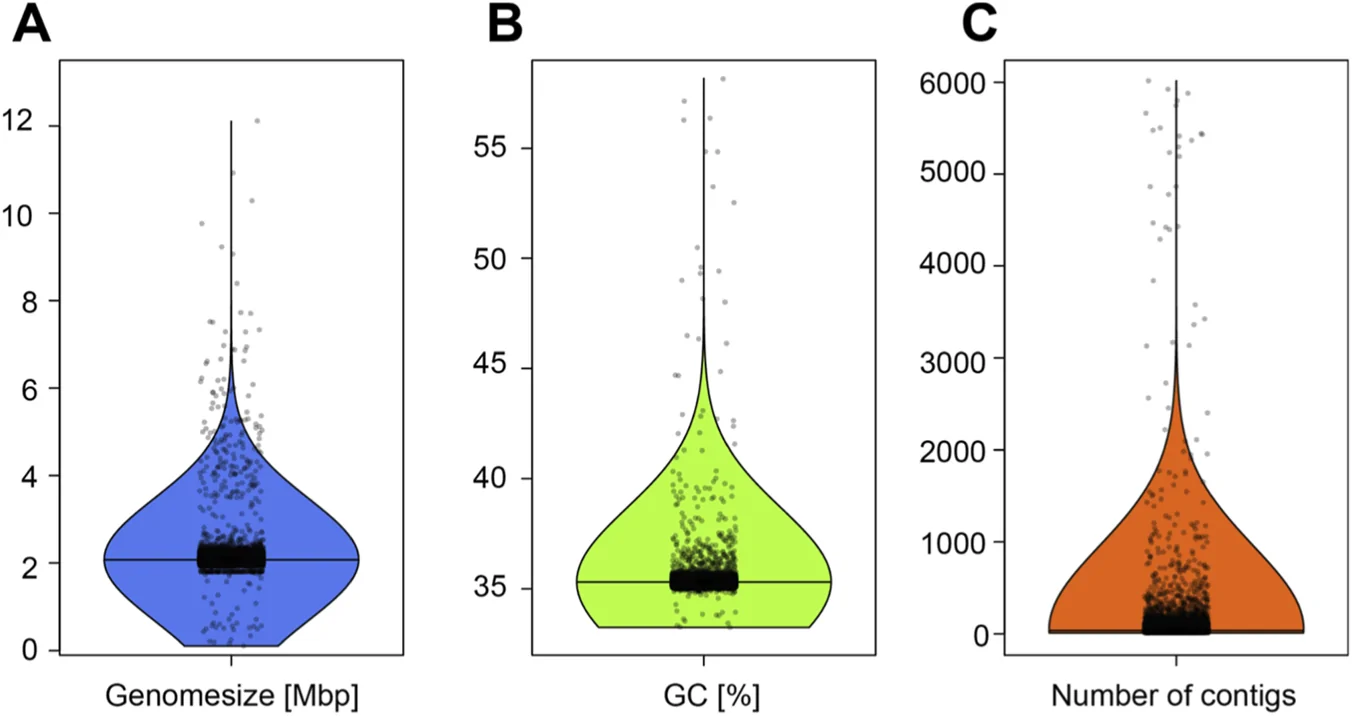
Comparison of genome size **(A)**, GC content **(B)**, and number of contigs **(C)** of all GBS genomes contained in BakRep v2 (n = 37,970). Individual dots represent single genomes, with horizontal jitter added to avoid overplotting. Horizontal black lines indicate medians; bold black bars represent interquartile ranges; vertical black lines represent outliers.

### Metadata of GBS genomes

The genomes originate from 51 countries across six continents, with a strong overrepresentation of North America (49.73%; n = 18,884) and of the United States in particular (48.59%; n = 18,451). No isolation region was provided for 14,747 (38.84%) genomes (Figure 2).

**FIGURE 2:**
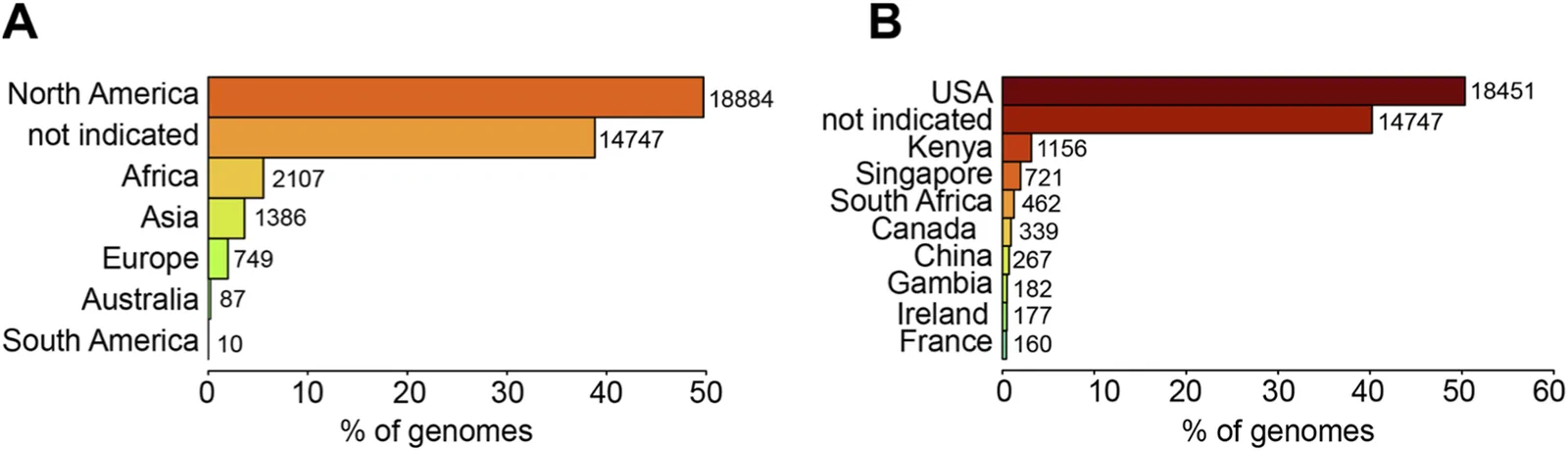
Distribution of GBS genomes contained in BakRep v2 across different continents **(A)** and the ten most common countries **(B)**. The total number of genomes for each country and continent, respectively, is displayed right to the bars.

Most genomes were declared as human isolates (58.41%; n = 22,179), followed by 61 bovine (0.16%) and 48 fish-derived genomes (0.12%). Three (0.01%) genomes were from dogs, and single genomes (0.004%, each) from a cat, a llama, and a crocodile. Host species was not provided for 41.28% (n = 15,675) of genomes. For 98.35% (n = 37,342) of the genomes, no information on disease status was provided; 0.88% (n = 334) were reported as diseased, 0.60% (n = 226) as healthy, and 0.16% (n = 60) as carriage isolates. The collection year spans from 1972 to 2024, although no year was provided for 39.05% (n = 14,829) of genomes. As complete dates (DD/MM/YYYY) were unavailable for the majority of records, and given the size of the dataset (n = 37,970), only the collection year was extracted and used for all temporal analyses. Genome counts increased gradually over time: Between 1972 and 2005 they remained very low, with fewer than 64 genomes per year (each <0.17% of the total dataset) but increased noticeably from 2006 onward. Most genomes were collected between 2015 and 2022, (each year accounting for >6.32% of the total dataset), peaking around 2019-2020, followed by a decline and only very few genomes for 2023 (0.32%; n = 120) and 2024 (0.06%; n = 24) (Supplementary Figure S2).

### Serotype, sequence type and clonal complex distribution

As serotype information was missing for 98.02% (n = 37,629) of genomes and the methodology used for serotype determination was unclear, capsular serotypes were *in silico* reanalyzed for all genomes. Most genomes belonged to one of the nine predominant GBS serotypes (75.33%) plus all four subtypes of serotype III (24.41%). Only 97 (0.26%) genomes were non-typeable. Serotype III was most common (24.41%; n = 9,270), followed by serotype Ia (21.07%; n = 8,000), and serotype V (16.25%; n = 6,172) (Figure 3A).

**FIGURE 3:**
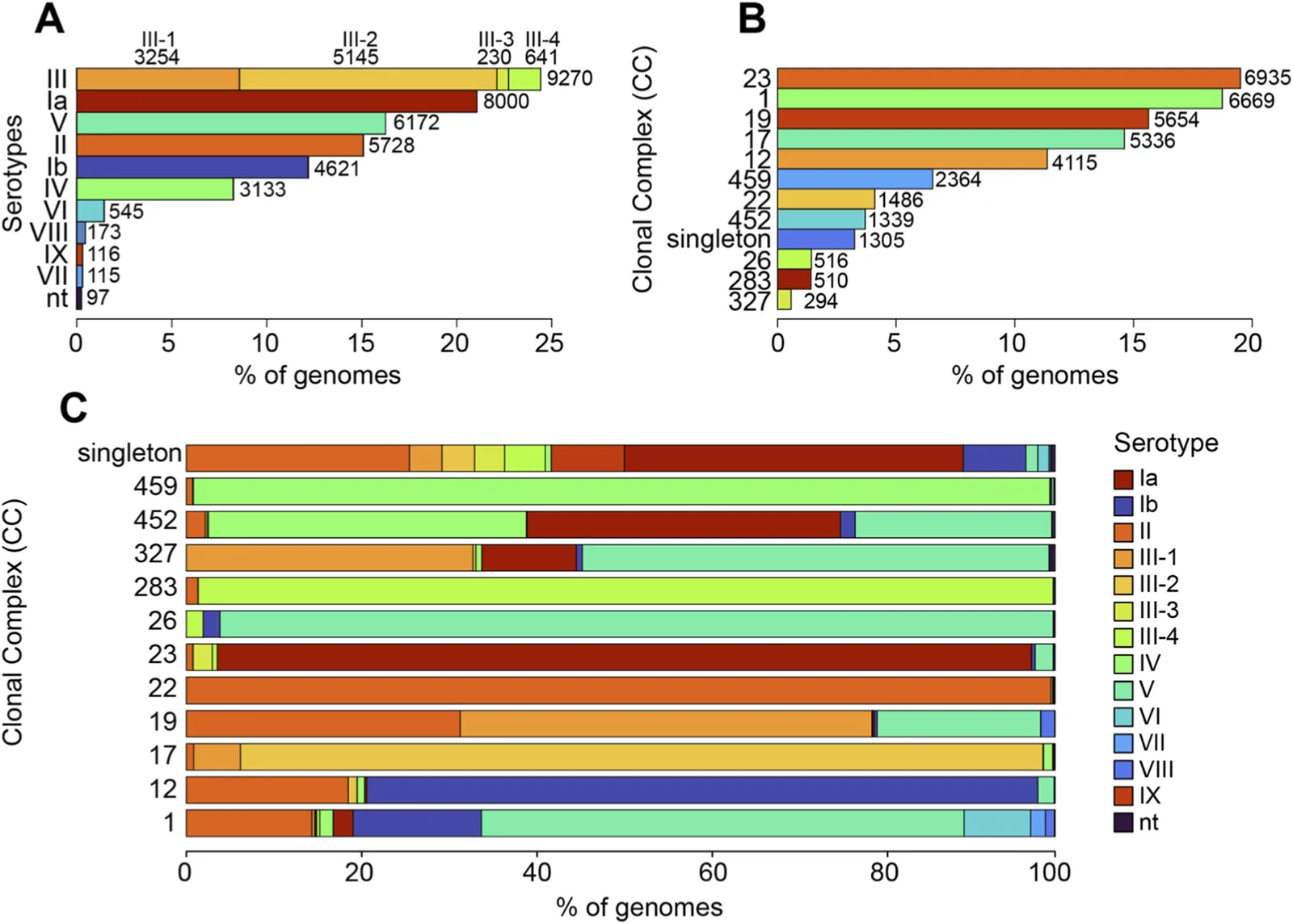
Serotype **(A)** and CC **(B)** distribution, and serotype allocation **(C)** among the CCs of all GBS genomes contained in BakRep v2. The total number of genomes for each serotype and CC, respectively, is displayed right to the bars.

MLST identified 1,015 unique STs, covering 40.39% STs currently listed in PubMLST. ST23 was most prevalent (14.56%; n = 5,530) of all genomes, followed by ST1 (14.31%; n = 5,434) and ST17 (11.62%; n = 4,414). STs grouped into eleven CCs, dominated by CC23 (18.99%; n = 6,935), CC1 (18.25%; n = 6,669), and CC19 (15.48%; n = 5,654) (Figure 3B). Serotype distributions within clonal complexes were assessed descriptively. For each clonal complex, genomes were cross-tabulated by predicted serotype, and proportions were calculated relative to the total number of genomes within the respective CC.

Serotypes were unevenly distributed across CCs (all percentages refer to the total number of genomes within the respective CC). Within CC23, 93.73% (n = 6,500) of genomes were serotype Ia, within CC17, 92.38% (n = 4,929) were serotype III-2, within CC459, 98.60% (n = 2,331) were serotype IV, and within CC22, 99.53% (n = 1,479) were serotype II. CC1 consisted predominantly of serotype V (55.56%; n = 3,705) but also comprised substantial proportions of serotypes Ib (14.78%; n = 986), II (14.42%; n = 962), and VI (7.67%; n = 512). CC19 showed a more even distribution across three serotypes, comprising III-1 (47.40%; n = 2,680), II (31.52%; n = 1782), and V (18.84%; n = 1,065) (Figure 3C).

Conversely, when percentages are calculated within serotypes, most serotypes occurred across multiple STs, whereas some were confined to few lineages: 93.10% (n = 108) of serotype IX genomes belonged to ST130, with the remainder found in ST1208 and ST1216 (both single-locus variants of ST130) and in ST24 (CC452). Serotype VI genomes were almost entirely restricted to CC1, with two exceptions assigned to CC12 and CC16 (ST889). Similarly, serotype VII genomes were found almost exclusively within CC1, except for single genomes in ST103 and ST1438. Detailed numbers are provided in Supplementary Table S1.

### Continental and temporal variation in serotypes and clonal complexes

Geographic origin was available for 23,223 of the 37,970 genomes (61.16%). The remaining 14,747 genomes (38.84%) lacked this information and were therefore not included in the continental analysis. Unless stated otherwise, percentages in this section are calculated relative to the relevant reference group (e.g., the total number of genomes from a given continent). Serotype and CC distributions across continents and collection years were assessed by descriptive cross-tabulation, with proportions calculated relative to the total number of genomes from the respective continent or year. Because the dataset does not derive from structured or representative sampling and the number of genomes differs substantially between regions and years, no statistical comparisons between geographic regions or time periods were performed. All reported geographic and temporal patterns are therefore descriptive. Serotype and CC distributions varied geographically (Figure 4). In North America, serotypes Ia (20.74%; n = 3,917), III (18.43%; n = 3,480; across all subtypes), and II (17.90%; n = 3,380) were most frequent (Figure 5A). In Africa serotype III (42.76%; n = 901) predominated, particularly subtypes III-1 and III-2 (Figure 5B), while Asia showed a distinct pattern dominated by serotype III-4 (28.14%; n = 390), a subtype that was rare in other regions (Figure 5C). In Europe, serotypes III-2 (24.03%; n = 180), Ia (20.83%; n = 156), and II (18.02%; n = 135) were most common, whereas in Australia, serotype Ib (29.89%; n = 26) was most frequent albeit based on a very small number of genomes (Figures 5D,E). For CCs, CC1 (20.68%; n = 3,763) and CC23 (19.78%; n = 3,599) dominated in North America, CC17 in Africa and Europe (30.75%; n = 643 and 32.86%; n = 230), and CC283 (28.11%; n = 377) and CC1 (27.89%; n = 374) in Asia. Notably, both serotype III-4 and CC283 were found almost exclusively in Asia, although the limited number of Asian genomes precludes firm conclusions about their true regional prevalence.

**FIGURE 4:**
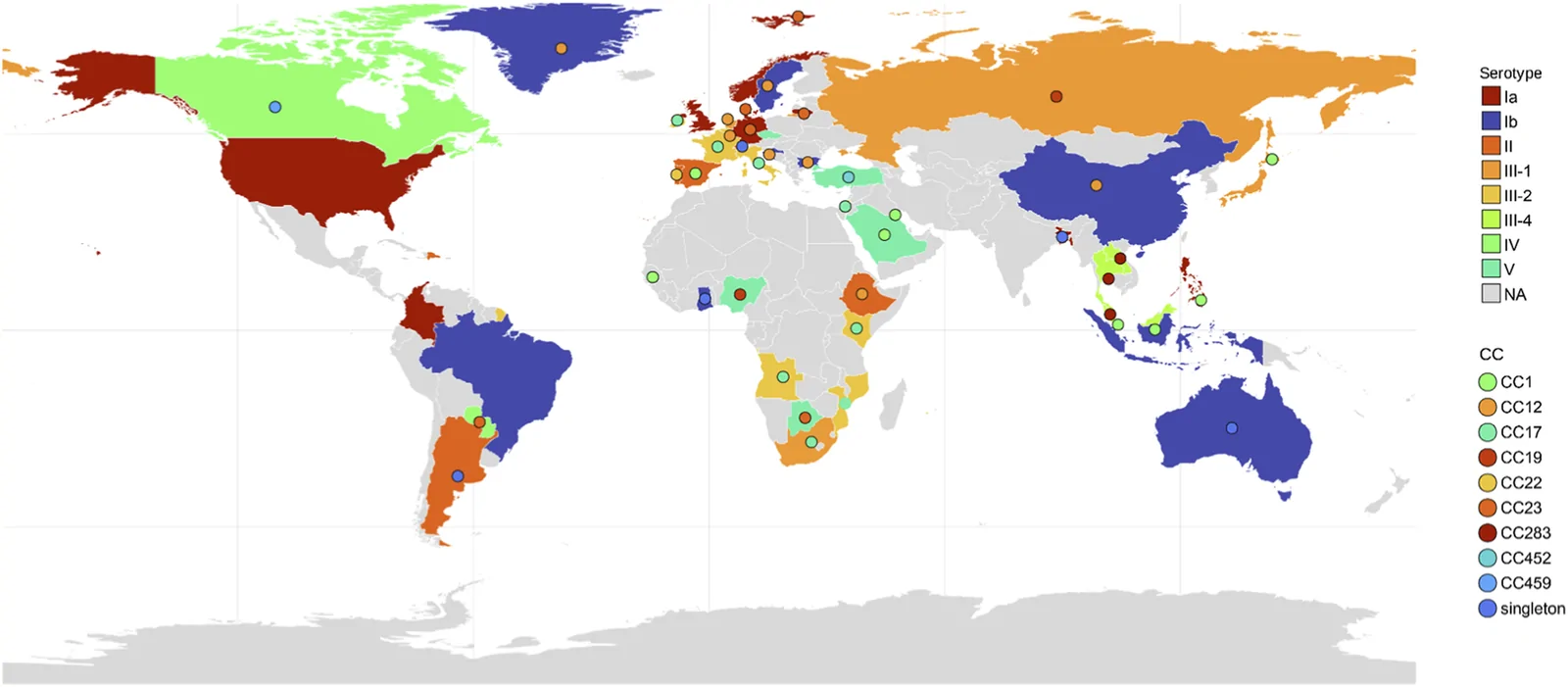
Serotype and CC distribution by region. The color of each country represents the most prevalent serotype, while the colored circle indicates the dominant CC.

**FIGURE 5:**
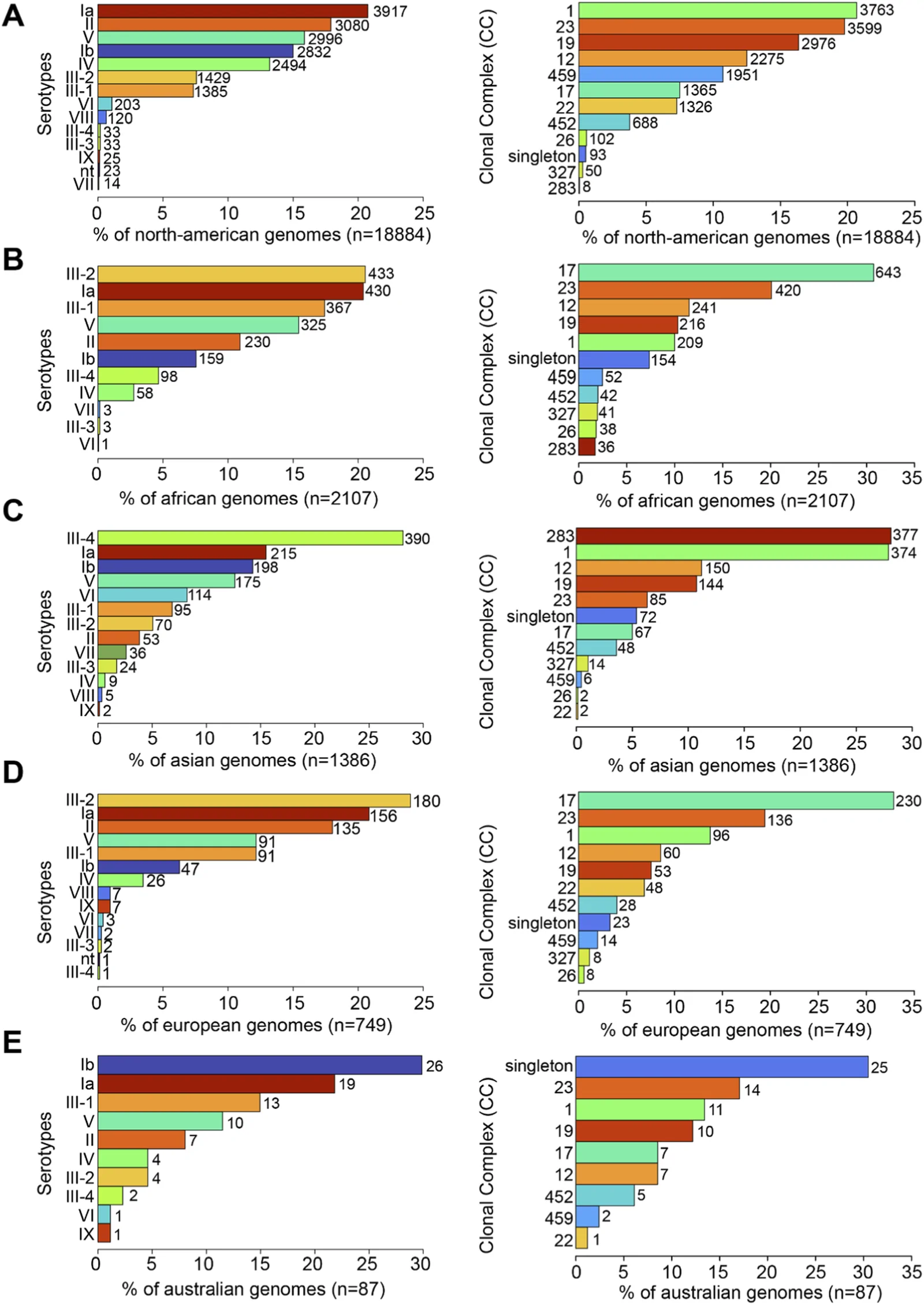
Serotype (left) and CC (right) distribution by continent. **(A)** North-American genomes, **(B)** African genomes, **(C)** Asian genomes, **(D)** European genomes and **(E)** Australian genomes. The total number of genomes is displayed right to the bars.

Serotype and CC composition varied across the study period, but the number of genomes available per year differed by several orders of magnitude (Supplementary Figure S2), which strongly constrains interpretation of these patterns. Serotype Ia has remained consistently prevalent, as did serotype V, though with greater fluctuations. Serotypes Ib and II increased gradually, III-1 declined slightly, and III-3, VIII, and IX remained at consistently low frequencies (Figure 6A). The number of distinct CCs observed per year increased in parallel with the number of sequenced genomes. Prior to the early 1990s, CCs were largely restricted to CC1 additional CCs (CC17, CC19, CC23, and CC283) emerged from the mid-1990s at low frequencies and from 2004 onward, multiple lineages (CC1, CC12, CC17, CC19, and CC23) co-occurred and increased rapidly (Figure 6B). As early years are represented by very few genomes, the geographic composition of the dataset varies over time, and US-derived genomes account for approximately half of all genomes, the temporal trends described are strongly influenced by sampling and sequencing activity in North America and cannot be interpreted as global epidemiological trends. Detailed numbers are listed in Supplementary Table S2.

**FIGURE 6:**
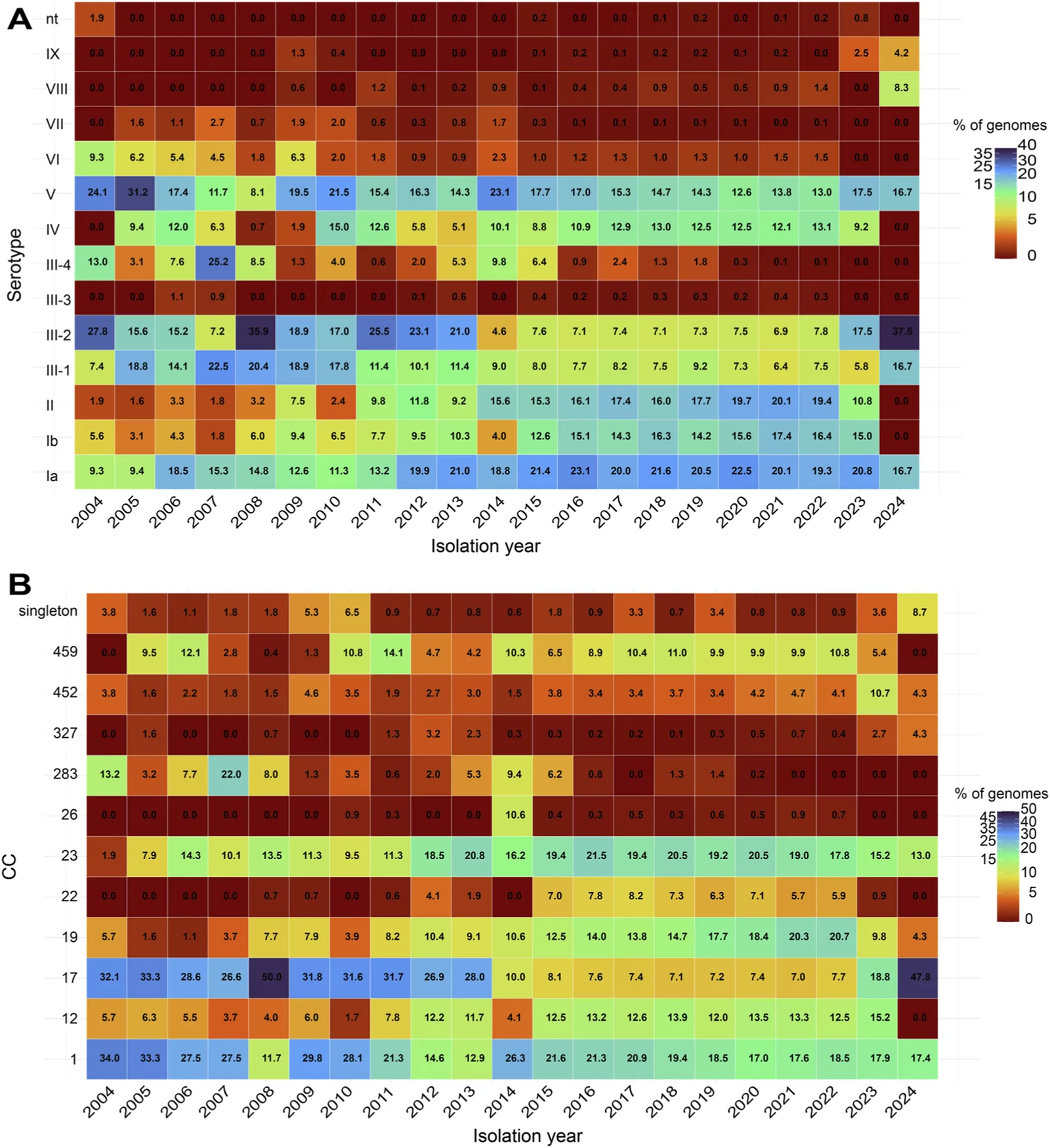
Serotype **(A)** and CC **(B)** distribution per year. Displayed are the years 2004–2024. The heatmap shows the percentage of genomes assigned to each serotype and CC for each isolation year. Color intensity represents the proportion of genomes within each year.

### Resistome of GBS genomes

At least one AMR gene was detected in 88.10% (n = 33,452) of genomes. In total, 132 AMR determinants conferring resistance against 24 drug classes were identified. Tetracycline resistance genes (16 different) were most prevalent (84.24%; n = 31,985), mainly *tet(M)* (76.99%; n = 29,232) and *tet(O)* (8.42%; n = 3,196). Macrolide-lincosamide-streptogramin B (MLSB) resistance genes were the second-most common (17.66%; n = 6,707), predominantly *erm(B)* (16.38%; n = 6,220), *erm(A)* (13.11%; n = 4,976) of genomes, and *erm(T)* (1.3%; n = 492). Aminoglycoside resistance genes were found in 4.15% (n = 1,574) and beta-lactam resistance genes in only 0.14% (n = 55) of genomes.

More than one AMR gene was detected in 41.34% (n = 15,695) of genomes, including 447 genomes with more than five and 11 genomes with more than ten hits. Genes for multiple drug classes occurred in 40.57% (n = 15,403), and 2.89% (n = 1,098) carried genes for three or more classes, indicating multidrug resistance. Notably, one genome harbored resistance genes against seven drug classes (ID: SAMEA112769107).

As erythromycin and tetracycline resistance genes frequently co-localize on the same transposons, we examined their co-occurrence. Both were present in 27.62% (n = 10,485) of genomes, whereas a tetracycline resistance gene alone occurred in 56.62% (n = 21,500), and an erythromycin resistance gene alone in 2.92% (n = 1,109). The most common combinations were *erm(B)/tet(M)* (1.1%; n = 4,180), *erm(A)/tet(M)* (1.04%; n = 3,963), and *erm(B)/tet(O)* (0.58%; n = 2,209). T*et(M)/mef(A*) was detected in 7.86% (n = 2,979), and the combination of *tet(O*)/*mef(A)* in 0.48% (n = 183) of genomes. As macrolide efflux in streptococci is mediated by *mef(A)* (transmembrane channel) and *msr(D)* (ATP-binding domains) (Iannelli et al., 2018), we assessed both genes. They co-occurred in 8.52% (n = 3,236) of genomes, while *mef(A)* alone occurred in 0.04% (n = 16) and *msr(D)* alone in 0.01% (n = 4). Detailed numbers are provided in Supplementary Table S3.

### Serotype and CC associated resistome patterns

Among the eleven CCs, CC1 harbored the highest number of distinct AMR genes (n = 72) (Figure 7B). *Tet(M)* was the most prevalent gene in all CCs (Figure 7), except CC459, which was instead characterized by a markedly high frequency of *erm(A)* (Figure 7F). Aminoglycoside resistance genes (e.g., *ant* (Danmallam and Pimenov, 2019)*-Ia, aph(3′)-IIIa)* occurred in all CCs, most abundantly in CC17 (Figure 7D) which also showed the highest number of hits for the trimethoprim resistance gene *dfrF*. Several vancomycin resistance genes were exclusively found in CC1 (*vanS-G, vanY-G1, vanR-G, vanU-G*), while *msr(D)* was by far the most common in CC23 (Figure 7A).

**FIGURE 7:**
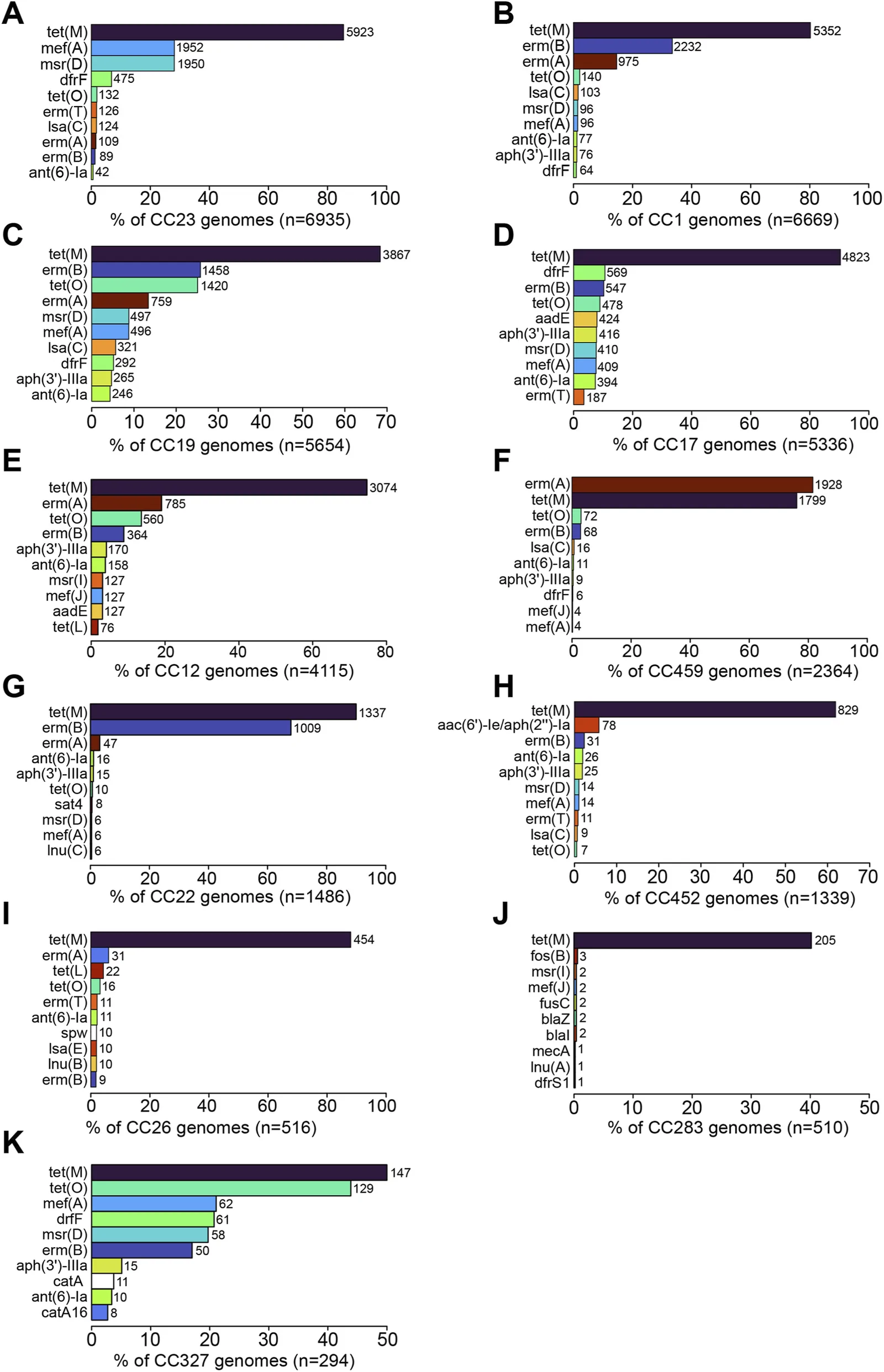
Distribution of the ten most prevalent AMR genes per CC. **(A)** CC23, **(B)** CC1, **(C)** CC19, **(D)** CC17, **(E)** CC12, **(F)** CC459, **(G)** CC22, **(H)** CC452, **(I)** CC26, **(J)** CC283, **(K)** CC327. The total number of genomes for each AMR gene is displayed right to the bars.

Serotype II showed the highest AMR gene diversity (72 determinants), with *tet(M)* being universally present. In serotype VIII, *tet(O)* was more prevalent than *tet(M)*, and like CC459, serotype IV showed a high prevalence of *erm(A)*. In contrast, serotype Ia exhibited fewer *erm(A)* and *erm(B)* hits but a higher occurrence of *mef(A)* and *msr(D)* (Figure 8). The highest proportion of genomes without any AMR hit was observed in CC283 (58.82%), and CC452 (31.76%), and among serotypes in IX (95.69%), VIII (78.03%), VI (61.16%), and III-4 (51.34%).

**FIGURE 8:**
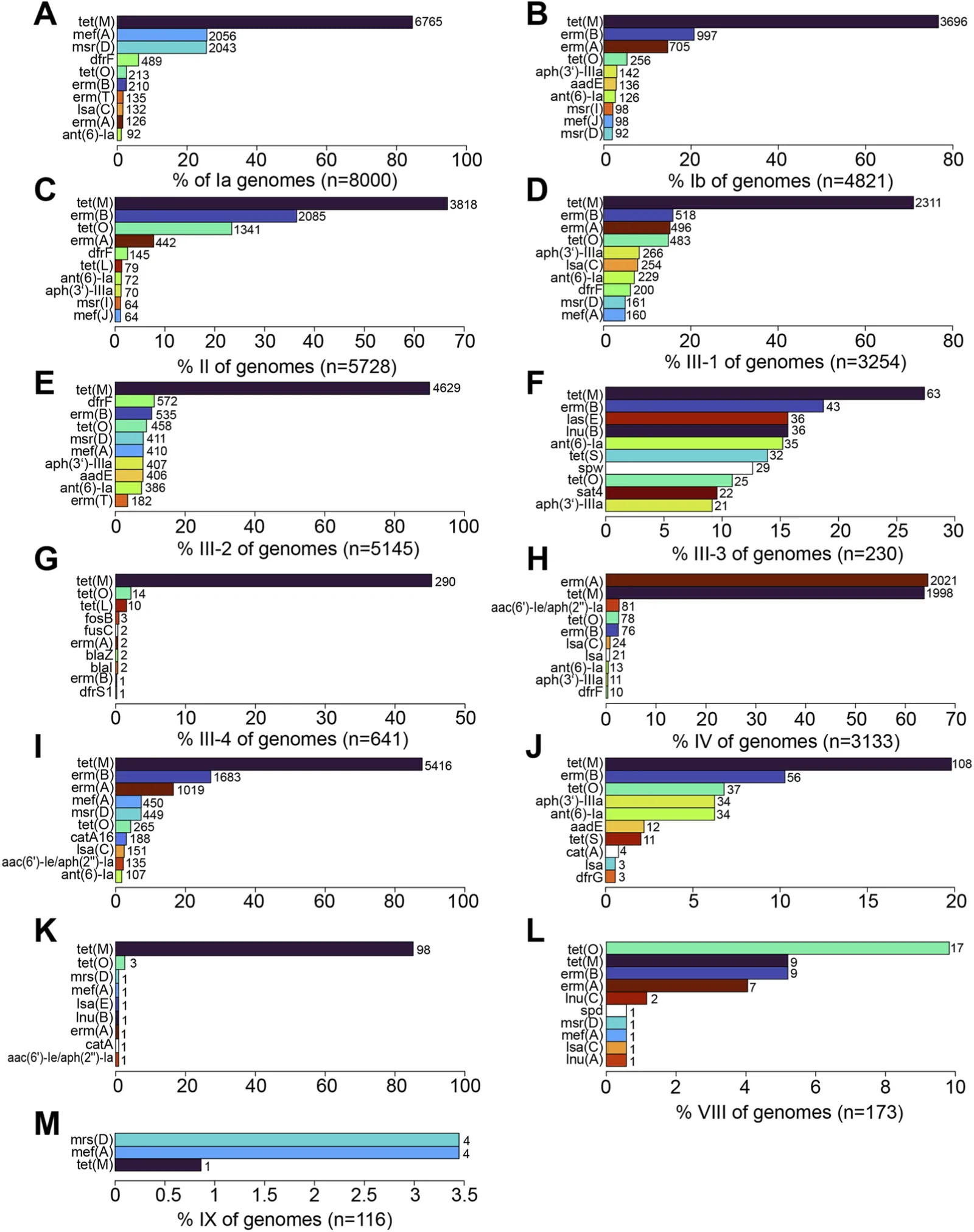
Distribution of the ten most prevalent AMR genes per serotype. **(A)** Ia, **(B)** Ib, **(C)** III-1, **(D)** III-2, **(E)** III-3, **(F)** III-3, **(G)** III-4, **(H)** IV, **(I)** V, **(J)** VI, **(K)** VII, **(L)** VIII, **(M)** IX. The total number of genomes for each AMT gene is displayed right to the bars.

### Regional resistome patterns

The geographical distribution of AMR genes was also assessed. *Tet(M)* was the most widespread gene, detected across all continents. North America exhibited the highest diversity and prevalence of AMR genes (n = 59), particularly macrolide and tetracycline determinants (Figure 9A), which likely reflects the strong overrepresentation of North American genomes rather than a truly higher regional burden. In Africa, several less common genes were identified, including *dfrF*, *aac(6′)-Ie/aph(2″)-Ia*, *msr(I)*, *mef(J)*, and *catA16* (Figure 9B), while resistance profiles in Asia and Europe were largely similar, albeit with slightly lower overall prevalence (Figures 9C,D). Given the uneven regional representation, these differences should be interpreted as descriptive only.

**FIGURE 9:**
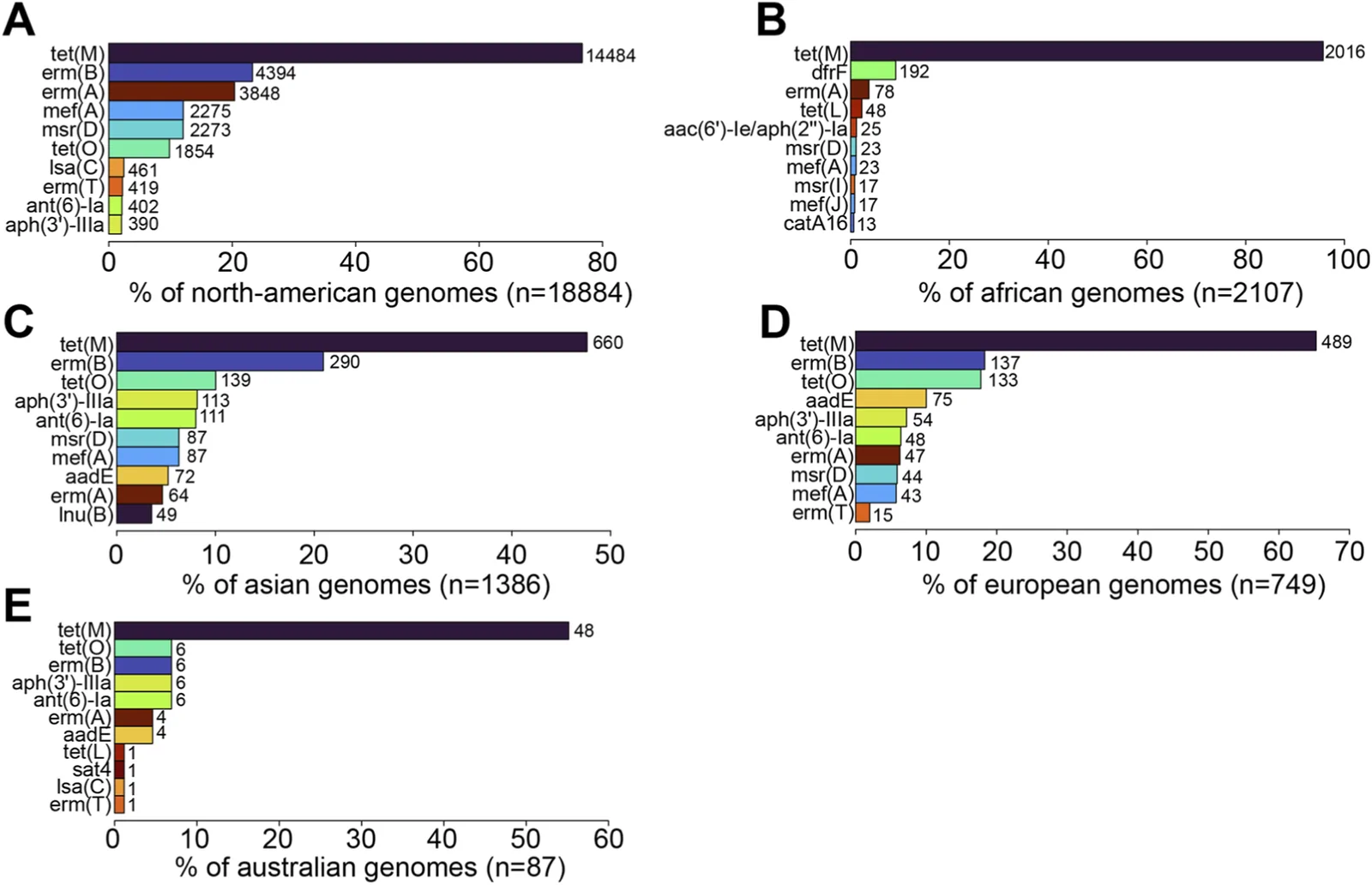
Distribution of the ten most prevalent AMR genes per continent. **(A)** North-America, **(B)** Africa, **(C)** Asia, **(D)** Europe, **(E)** Australia. The total number of genomes for each AMR gene is displayed right to the bars.

### Temporal resistome variations

AMR genes were largely absent before the 1990s, with only sporadic tetracycline and macrolide determinants in the late 1980s. *Tet(M)* remained the most prevalent gene over the past two decades, while *tet(O)* increased markedly from 2008 onward. MLSB genes such as *erm(A)* and *erm(B)* became more frequent from 2000 onward, whereas aminoglycoside resistance genes showed only sporadic peaks without a consistent trend. Overall, AMR genes increased in diversity and frequency over the last 20 years (Figure 10). Detailed numbers are listed in Supplementary Table S2.

**FIGURE 10:**
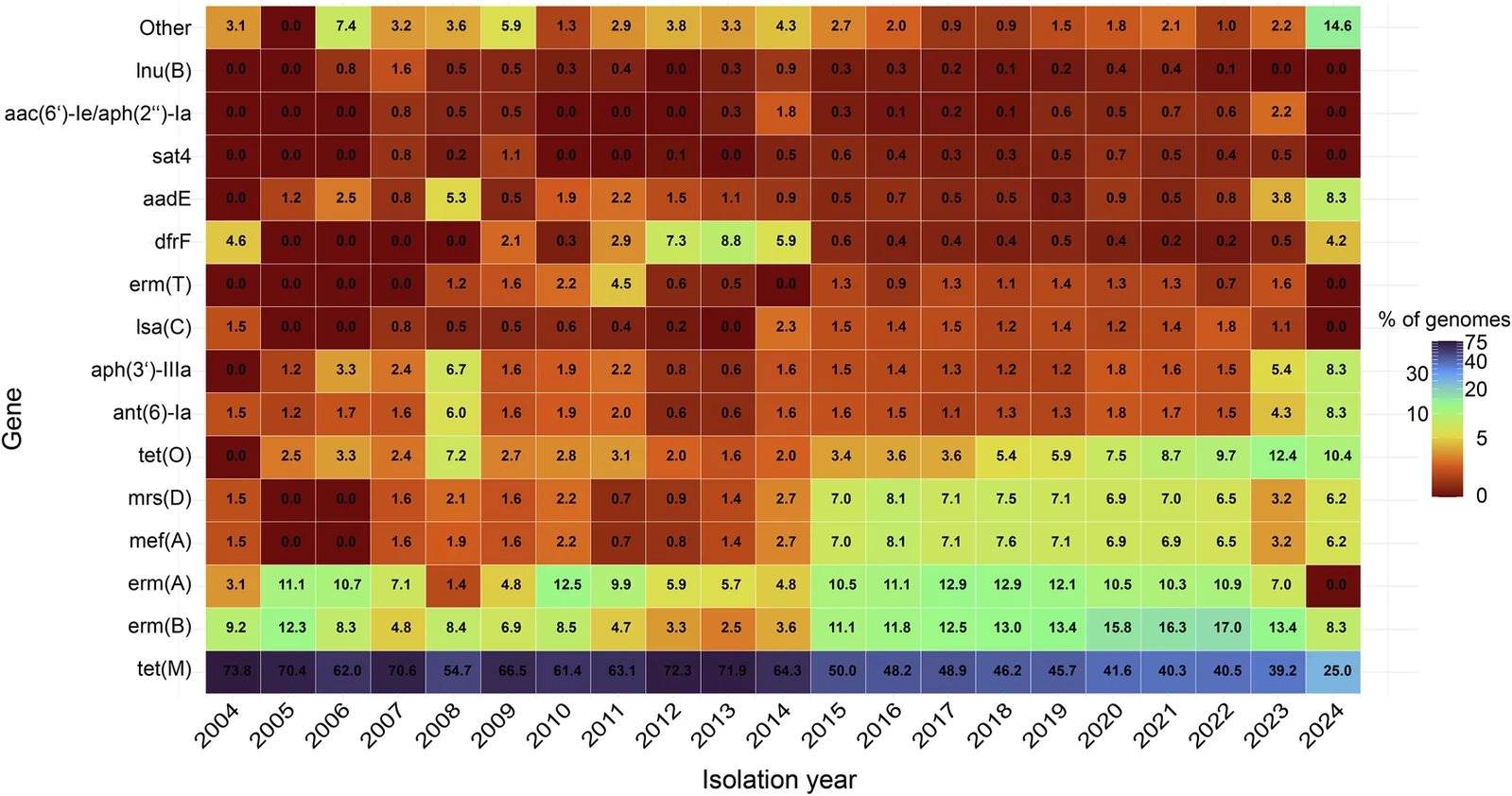
AMR gene distribution per year. Displayed are the years 2004–2024. The heatmap shows the percentage of genomes assigned to each gene for each isolation year. Color intensity represents the proportion of genomes within each year.

### Pan genome analysis and genome-wide association investigation

Gene-content analysis of the 37,970 genomes identified a pan genome of 274,610 genes and a core genome of 1,398 genes. Most genes occurred in only a small fraction of genomes, with more than 99% of genes present in fewer than 15% of isolates (cloud genes). We therefore filtered the Panaroo gene presence/absence matrix to retain genes present in >0.25% and <95% of genomes, yielding 4,101 genes (Figure 11). Phylogenetic analysis showed partial clustering by serotype and CC, alongside regions of highly diverse distribution (Figure 12).

**FIGURE 11:**
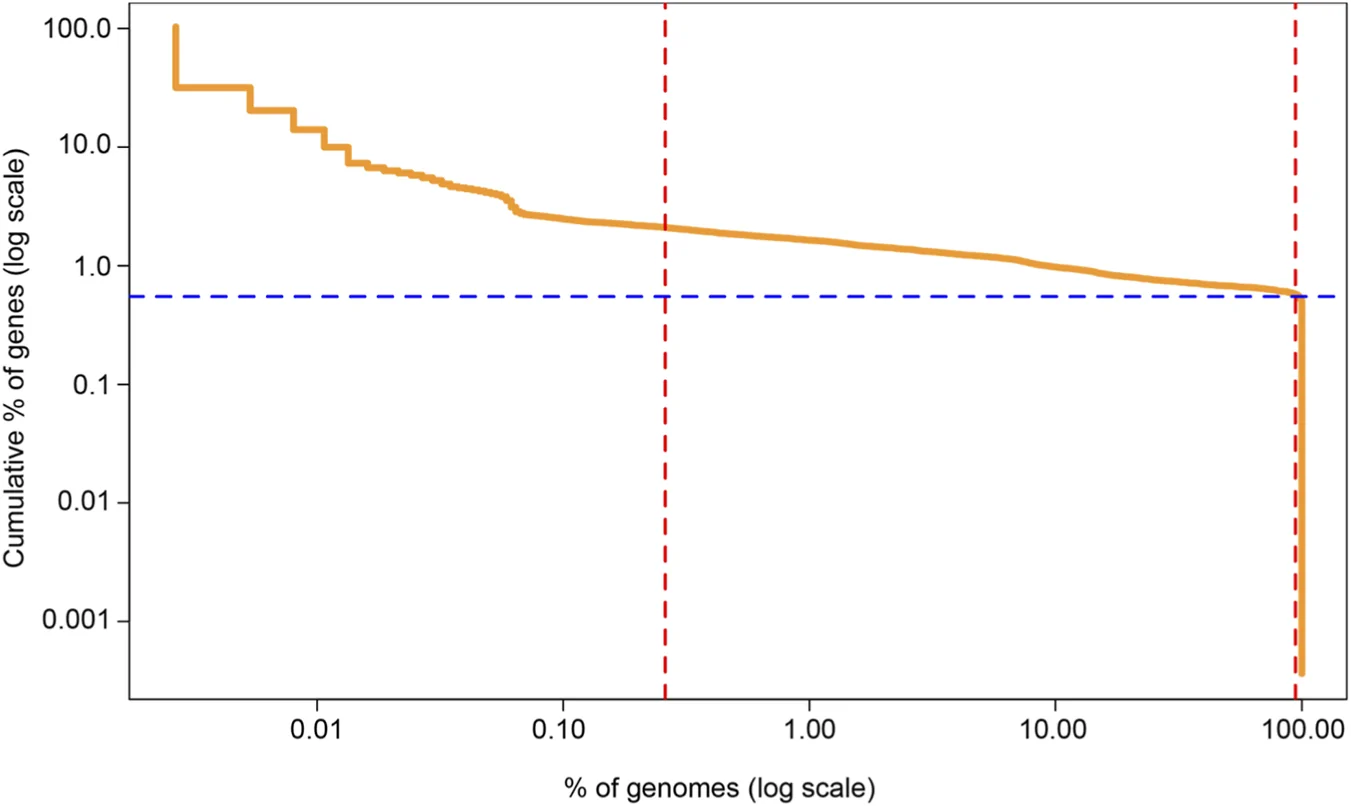
Pan-genome gene frequency distribution. The x-axis represents the percentage of genomes containing a given gene, and the y-axis represents the cumulative percentage of genes, both on logarithmic scales. Vertical red dashed lines indicate the lower and upper frequency cutoffs (0.25% and 95% of genomes), while the blue dashed horizontal line marks the core genome.

**FIGURE 12:**
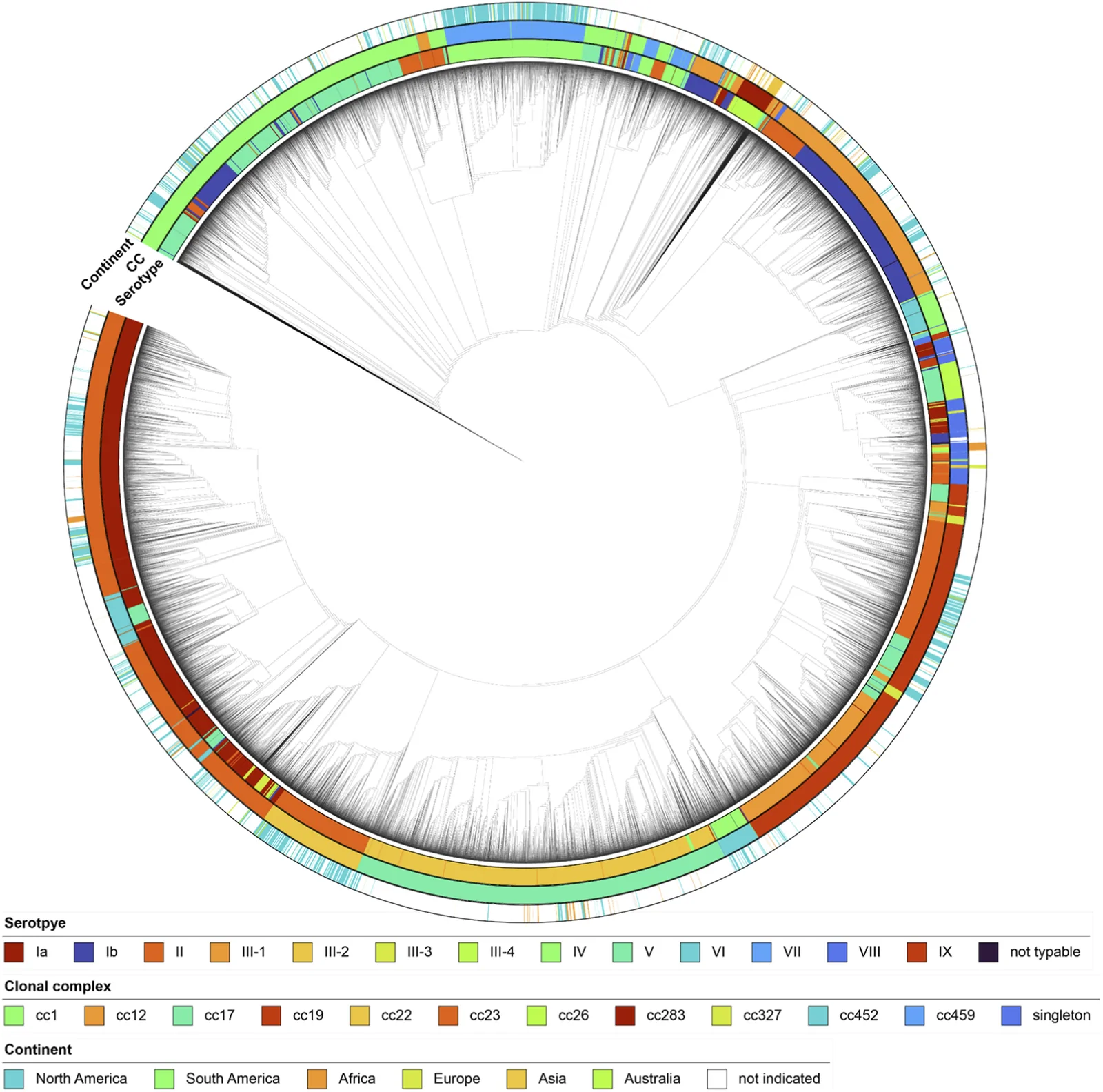
Phylogenetic tree of 37,970 GBS genomes. The tree was constructed with Mashtree. Colored blocks show serotype, clonal complex and continent of isolation. Annotation of the tree was done using iTol.

Scoary2 analyses were performed on the five most common serotype/clonal-complex combinations (Ia/23, II/22, III-2/17, IV/459, V/1). Genes were considered associated at >70% specificity and sensitivity, with 60, 54, 41, 86, and 71 genes positively and 31, 12, 17, and 1 genes negatively associated with Ia/23, II/22, III-2/17, IV/459, and V/1, respectively. Group III-2/17 had the most positively associated genes at >90% specificity and sensitivity (n = 30), followed by group II/22 (n = 17). Based on their functional annotations, the associated genes were grouped into broad functional categories. Ia/23 harbored several mobile genetic elements, numerous phage-associated genes, and loci involved in metal homeostasis and heavy-metal resistance, whereas II/22 and III-2/17 encoded more functionally specialized genes. II/22 was enriched for genes related to metal uptake, including siderophore biosynthesis, while III-2/17 showed a marked accumulation of virulence and adhesion determinants, particularly components of the accessory secretion system (SecA2/SecY2 and Asp proteins). IV/459 and V/1 were dominated by phage-associated genes. The top three positive and negative hits per group are shown in Table 1. All other genes are listed in Supplementary Table S4.

**TABLE 1:** Scoary2 results are reported for the most significant positively (+) and negatively (−) associated genes of the five groups ((Ia/23, II/22, III-2/17, IV/459, V/1).

Phenotype	pos (+)/neg (−)	Gene	Annotation	SE	SP	*p*-value
Ia/23 (n = 6,497)	+	group_40999	Phosphoribosylaminoimidazole carboxylase ATPase subunit	100.0	91.71	0.0020
	+	group_51961	DUF6287	100.0	91.69	0.0020
	+	*purN*	Phosphoribosylglycinamide formyltransferase	99.98	88.82	0.0020
	-	group_9603	GNAT family N-acetyltransferase	99.45	94.18	0.0020
	-	*aprE*	S8 family serine peptidase	99.46	85.12	0.0020
	-	group_11313	Hypothetical protein	99.86	73.96	0.0020
II/22	+	group_41231	DUF6261	100.0	98.61	0.0020
	+	group_52008	Membrane protein	100.0	98.60	0.0020
	+	group_51962	Phage protein	100.0	98.60	0.0020
	-	group_6272	Cingulin	100.0	81.76	0.0020
	-	group_23506	Tandem five-TM protein	100.0	73.78	0.0020
	-	group_19754	KTSC domain-containing protein	100.0	73.67	0.0020
III-2/17	+	group_6225	Cell wall surface anchor protein	99.98	90.46	0.0020
	+	spaA∼∼∼inlB	Sortase	99.96	90.31	0.0020
	+	group_10458	S8 family serine peptidase	99.94	96.21	0.0020
	-	group_41244	HIT family protein	99.96	74.90	0.0020
	-	group_9833	Lipoprotein	99.88	87.97	0.0020
	-	group_9933	TIGR04197 family type VII secretion effector	99.88	71.98	0.0020
IV/459	+	group_9154	Integrase	99.96	98.95	0.0020
	+	*padR*	Transcriptional regulator	99.96	98.95	0.0020
	+	group_41269	N-acetyltransferase	99.96	98.95	0.0020
	-	group_11745	Relaxase	99.96	77.27	0.0020
V/1	+	neuA∼∼∼wcaA∼∼∼cpsJ	Glycosyltransferase (capsular polysaccharide biosynthesis protein)	98.49	91.80	0.0020
	+	group_25544	Phage protein	98.22	76.39	0.0020
	+	group_24863	Phage protein	98.22	76.20	0.0020

The top 3 hits positive and negative are displayed. SE: Sensitivity of using the presence/absence of this gene as a diagnostic test to determine trait-positivity. SP: Specificity of using the absence/presence of this gene as a diagnostic test to determine trait-negativity. P-value: The *p*-value of the post-hoc permutation for the best gene.

As the *scpB-lmb* transposon has been reported mainly in association with the human host (Franken et al., 2001; Crest and ani, 2021), and most genomes were decelerated as human isolates, we examined its prevalence. S*cpB* was detected in 99.91% (n = 37,934) of genomes and *lmb* in 94.85% (n = 36,013). *ScpB* war represented by 55 distinct variants in Panaroo, whereas *lmb* occurred as a single variant.

## Discussion

*Streptococcus agalactiae* is a multi-host pathogen, primarily linked to infections in humans and cattle. Most studies focus on specific regions or outbreaks, leaving a lack of global perspective on GBS diversity. BakRep, with its scale and integration of genomic data and metadata, offers an opportunity to study broader trends and support more holistic analyses of the GBS population. Based on this repository, 37,970 GBS genomes were analyzed.

The predominance of North American genomes reflects the region’s leading role in clinical trials (44.19% of the market in 2024) (Marktgröße für klinische Studien, 2026). More striking is the absence of an isolation location in over a third of the data. The sharp rise in available genomes after 2006 likely reflects the introduction of the first commercially available DNA sequencing platform in 2005 (Margulies et al., 2005). Yet, as with the location, the isolation date is unknown for nearly two-fifths of genomes. To improve FAIR usability, the International Sequence Database Collaboration (INSDC) introduced standards requiring spatio-temporal metadata for new ENA submissions, but this has only been mandatory since May 2023 and still permits missing values (ENA, 2026). The same goes for the host species and the disease status (ENA Browser, 2026). The surge in submissions driven by cheaper and faster sequencing has outpaced the ability to check metadata quality (Price et al., 2024). Without adequate metadata, meaningful comparisons and global context become impossible, undermining transparency, reproducibility, and the utility of sequence data (Wilson et al., 2021).

Although overall GBS colonization rates are similar worldwide, the distribution of serotypes and sequence types varies by region, host species, and clinical presentation. Two global reviews reported serotype III as the most prevalent, followed by Ia and V (Le Doare and Heath, 2013; Bianchi-Jassir et al., 2020). When serotype III subtypes are combined, this pattern mirrors our findings. Serotype III is predominantly linked to neonatal meningitis (Hsu et al., 2021; Davies et al., 2004), while Ia and V are more often associated with invasive infections in non-pregnant adults (Alhhazmi et al., 2016; Farley and Strasbaugh, 2001). As over half of our genomes are human-derived, this distribution is expected, though it remains unclear whether it holds across non-human hosts. Serotype IX, only recently identified (Slotved et al., 2007), is already common in certain regions and serotype IV warrants increasing attention as a hotspot for recombination across diverse genetic backgrounds (Shabayek and Spellerberg, 2018). This growing diversity, combined with capsular switching, poses a major challenge for capsular polysaccharide-based vaccines, which may exert selective pressure enabling escape variants. Such switching from serotype III to IV within the hypervirulent CC17 lineage has already been demonstrated (Bellais et al., 2012; Meehan et al., 2014). Consistently, CC23, CC1, and CC19 are reported as the most frequent, widely distributed global lineages (Manning et al., 2008; Jones et al., 2003; Springman et al., 2014).

Our data confirm several well-established serotype-lineage associations. CC17 is almost exclusively linked to III-2, with III-2/ST17 representing a particularly virulent clone, associated with late-onset neonatal disease (Jones et al., 2003; Tong et al., 2007; Kao et al., 2019; Tsai et al., 2019; Kong et al., 2003). We confirm the strong III-2/CC17 association, although disease status cannot be assessed due to missing metadata. Similarly, III-1/ST19 is well known in early-onset neonatal disease and colonizing isolates (Davies et al., 2004; Tong et al., 2007; Sun et al., 2005), andCC23/Ia is strongly linked to invasive disease in non-pregnant adults (Meehan et al., 2014; Cubria et al., 2021). CC459 stood out for a high prevalence of the macrolide resistance gene *erm(A)*, clinically relevant given its strong association with serotype IV, which dominates adult disease (Teatero et al., 2015). The CC1/V link is also confirmed, with most serotype V isolates classified as ST1 and ST19 as the main non-ST1 background (Salloum et al., 2011; Flores et al., 2015).

Such strong serotype specificity suggests stable clonal lineages and conserved capsule types, useful as markers for targeted surveillance. In contrast, heterogeneous lineages such as CC1 and CC19, with CC1 showing the broadest serotype diversity, likely reflecting a diverse ancestral population rather than ongoing recombination (Jamrozy et al., 2023), complicate vaccination strategies and may harbor non-vaccine strains.

Regional patterns further illustrate these dynamics. Although serotype III is described predominant in Africa, data on the most common subtype remain scarce (Ntozini et al., 2025; Wadilo et al., 2023; Chukwu et al., 2015). Globally, III-1 and III-2 are the most frequent subtypes (Tong et al., 2007). Serotype III-4 and ST283 have been linked to fish disease in Asia (Delannoy et al., 2013; Ip et al., 2016), but more detailed host metadata are needed to distinguish a fish-associated lineage from zoonotic transmission. Serotype Ib, reported as more prevalent in Asia (Zhang et al., 2021), could not be directly confirmed, as it occurs at similar proportions in North America and predominates in Australia, albeit with very low genome numbers there.

AMR is an increasingly relevant concern in GBS. Penicillin remains first-line for colonized pregnant women, with clindamycin, erythromycin, and vancomycin as alternatives for penicillin allergy (Silva et al., 2023), but rising resistance to erythromycin and clindamycin has led to updated guidelines, recommending cephalosporins or vancomycin for adults (Khan et al., 2023). Only few genomes contained potential beta-lactam resistance genes, as beta-lactam resistance in GBS primarily involves penicillin-binding protein (PBP) substitutions (Bonofiglio et al., 2018), which was not addressed in the present analysis. Notably, MLSB resistance genes, particularly *erm(B)* and *erm(A)*, were the second-most prevalent drug class, an important trend for future research. Among aminoglycoside-resistant genomes, 340 harbored gentamicin resistance genes, relevant given the use of penicillin–gentamicin combinations for severe invasive infections (Khan et al., 2023).

Over 80% of genomes harbored tetracycline resistance genes. This well-known resistance (Hsu et al., 2025; Gergova et al., 2024) largely stems from widespread use of tetracycline since 1948, which likely promoted clonal expansion of resistant strains and the dissemination of the hypervirulent ST17 clone (Hayes et al., 2020; Da et al., 2014). Tetracycline resistance genes frequently co-localize with erythromycin resistance genes on mobile elements, with *erm(B)* often co-occurring with *tet(M)*, as also seen here (Hsu et al., 2025). While *tet(M)* predominates, elements carrying *tet(O)* or *tet(S)* have also been reported (Hayes et al., 2020; Pinto et al., 2014) and erythromycin resistance determinants are frequently linked to *tet* genes and conjugative transposons capable of horizontal transfer (Pinto et al., 2014). Such co-occurrence should be closely monitored as a potential indicator of multidrug-resistant lineages. Importantly, detection of AMR genes does not automatically imply phenotypic resistance, which requires gene expression and may be modulated by additional mutations. Genotypic results should therefore be interpreted cautiously and ideally supported by phenotypic testing.

Finally, distinct CC/serotype combinations are associated with characteristic gene repertoires spanning a continuum from genomic flexibility to niche-specific specialization, likely contributing to lineage-specific invasive potential. The enrichment of mobile genetic elements and phage-associated genes suggests enhanced genomic plasticity trough horizontal gene transfer, whereas II/22 shows focused enrichment of siderophore and metal-uptake genes, indicating specialization for iron-limited niches. III-2/17 accumulates virulence and adhesion genes, notably the accessory SecA2/SecY2 secretation system. This locus is largely restricted to the hypervirulent ST17 lineage encoding the Srr2 adhesin which mediates high-affinity binding to fibrinogen and plasminogen, enhancing adhesion, resistance to phagocytosis, and persistence in a murine meningitis model (Six et al., 2015). Its presence in group III-2/17 supports the reliability of our analysis and indicates that even a small set of accessory genes may strongly GBS adaptation.

Despite the scale of this dataset, several limitations must be considered. The data show a pronounced geographic and temporal bias, with a strong overrepresentation of North American genomes and of isolates collected after 2006, limiting the global generalizability of our findings. Importantly, this imbalance also confounds the temporal patterns described above: the number of genomes available per year increases by orders of magnitude across the study period, and absolute counts of virtually all serotypes and CCs rise accordingly. Such increases therefore primarily reflect the expansion of genome sequencing rather than changes in the underlying bacterial population. The temporal and geographical patterns reported here should therefore be regarded as descriptive of the sequenced collection rather than as representative of population-level epidemiology, Furthermore, substantial metadata gaps preclude a fully comprehensive evaluation: isolation location and date are missing for over a third of genomes, data for some hosts (e.g., camelids) appear entirely absent, and for most human isolates it is unclear whether they derive from adult or neonatal cases. As a result, associations between lineages and disease status could not be assessed. The continuous generation of sequencing data without adequately structured and curated metadata therefore limits the biological and clinical utility of such resources, representing a major obstacle in modern genomics and personalized medicine.

## Conclusion

In this large-scale analysis of 37,970 GBS genomes, we confirmed key serotype–lineage associations, including the strong link between III-2 and the hypervirulent CC17 clone, and identified distinct lineage-specific gene repertoires ranging from high genomic plasticity to niche specialization. We further observed widespread tetracycline resistance and a notable prevalence of MLSB resistance genes, underscoring the need for continuous AMR surveillance. Most importantly, our findings highlight that incomplete and non-standardized metadata critically limit the interpretation of such large genomic datasets. Safeguarding the quality and completeness of metadata is therefore essential to fully realize the potential of public sequence repositories for GBS research and beyond.

## Data Availability

All supporting data have been provided within the article or through the supplementary data. BakRep is accessible via https://bakrep.computational.bio/.
