# Supplementary material for: Towards a holistic epidemiology of Streptococcus agalactiae using the BakRep repository
Source: Front Bioinform. 2026 Sep 30;6:1924154. doi: 10.3389/fbinf.2026.1924154 (PMC13662052; doi:10.3389/fbinf.2026.1924154)
Supplement: Supplementary file 1 [file DataSheet1.pdf]

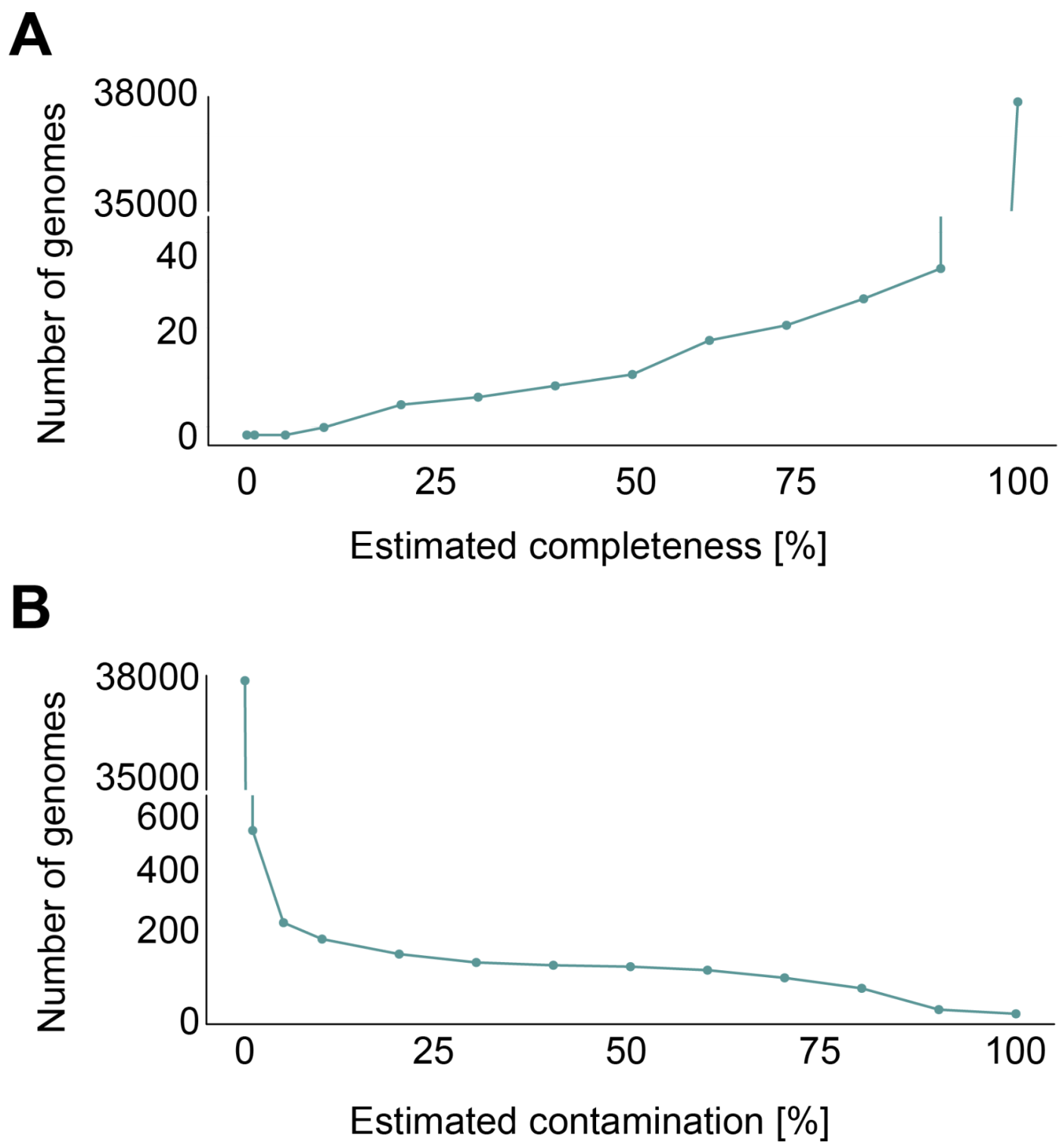

Supplemental Figure 1: Distribution of completeness **(A)** and contamination **(B)** level of all GBS genomes contained in BakRep v2.

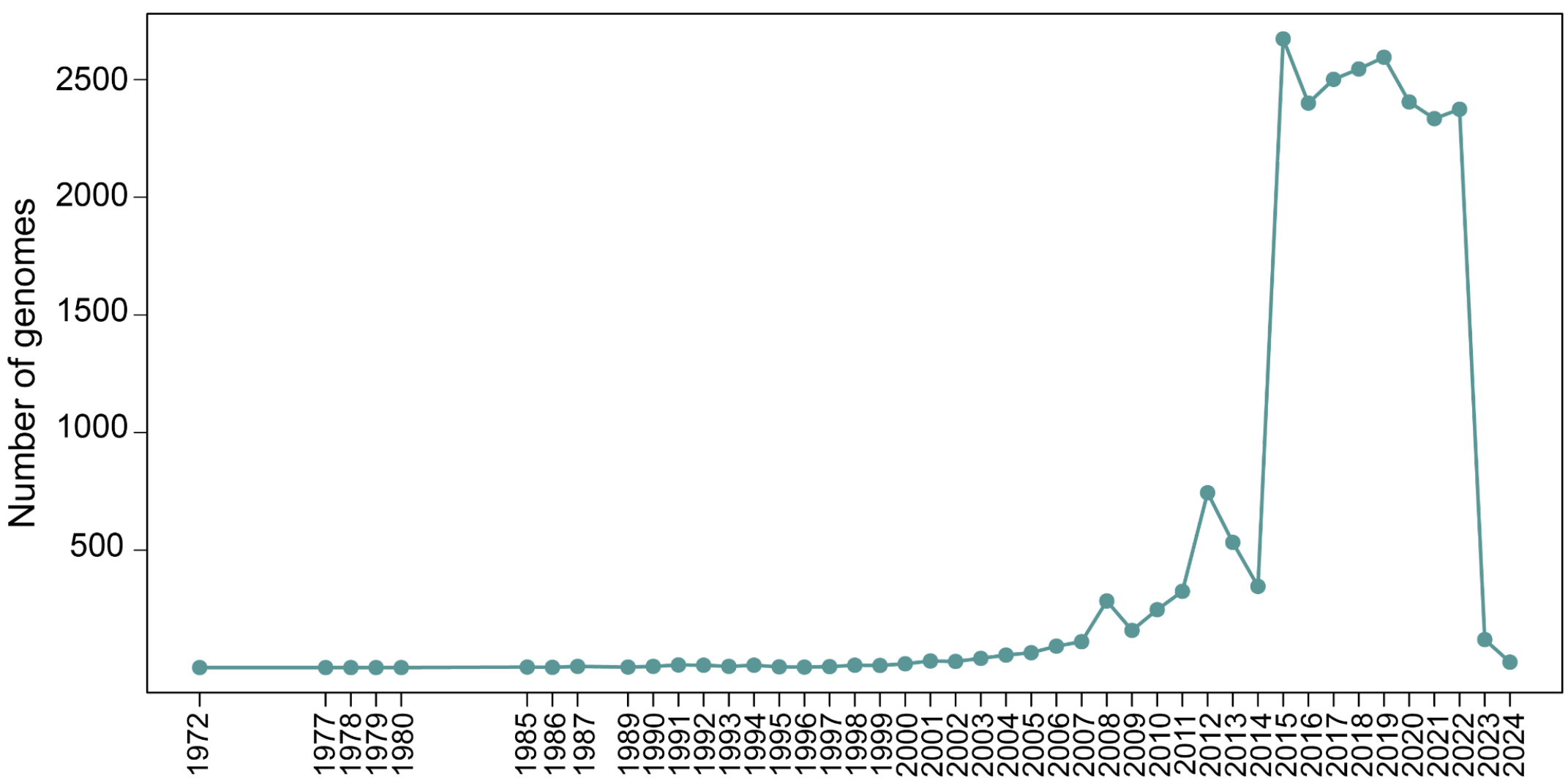

Supplemental Figure 2: The yearly rate of GBS genomes contained in BakRep v2. The linear chart shows the number of genomes per year from 1972 to 2024.
